# Attribution classification method of APT malware based on multi-feature fusion

**DOI:** 10.1371/journal.pone.0304066

**Published:** 2024-06-27

**Authors:** Jian Zhang, Shengquan Liu, Zhihua Liu

**Affiliations:** School of Computer Science and Technology, Xinjiang University, Xinjiang Uygur Autonomous Region, Urumqi, People’s Republic of China; Victoria University, AUSTRALIA

## Abstract

In recent years, with the development of the Internet, the attribution classification of APT malware remains an important issue in society. Existing methods have yet to consider the DLL link library and hidden file address during the execution process, and there are shortcomings in capturing the local and global correlation of event behaviors. Compared to the structural features of binary code, opcode features reflect the runtime instructions and do not consider the issue of multiple reuse of local operation behaviors within the same APT organization. Obfuscation techniques more easily influence attribution classification based on single features. To address the above issues, (1) an event behavior graph based on API instructions and related operations is constructed to capture the execution traces on the host using the GNNs model. (2) ImageCNTM captures the local spatial correlation and continuous long-term dependency of opcode images. (3) The word frequency and behavior features are concatenated and fused, proposing a multi-feature, multi-input deep learning model. We collected a publicly available dataset of APT malware to evaluate our method. The attribution classification results of the model based on a single feature reached 89.24% and 91.91%. Finally, compared to single-feature classifiers, the multi-feature fusion model achieves better classification performance.

## 1 Introduction

With the advancement of artificial intelligence, APT organizations are launching more complex attacks on computer systems using malware. In the current cyberspace environment, Advanced Persistent Threat (APT) [1] is one of the most representative attacks, and its persistent outbreaks have brought unprecedented security challenges [2]. Therefore, APT attacks have attracted high attention from many researchers and governments. APT attacks refer to individuals or organizations using advanced techniques to conduct long-term and persistent network attacks on specific targets. The difference between APT attacks and traditional network attacks lies in their characteristics of stealthiness, targeting, persistence, and organization [3]. APT attacks employ diverse attack techniques, yielding significant impact and being challenging to defend against, as demonstrated by the notorious Advanced Persistent Threat attack “Stuxnet” [4]. This virus emerged in 2010 and featured sophisticated and covert detection techniques, resulting in a lengthy discovery and analysis process. Moreover, the Stuxnet virus primarily targeted Iran’s nuclear facilities, significantly influencing their nuclear program. This incident is regarded as an organized state-sponsored act.

In traditional malware, the Windows API and its parameters provide information about the software’s access to system resources, thus revealing potential intentions [5]. The opcode represents a runtime instruction, and analyzing the opcode can provide insights into malicious intentions.

Like traditional attacks, APT attackers need to use malware as their attack weapon to launch cyber attacks [6]. Therefore, analyzing the malicious software used in APT attacks provides a feasible method for APT attack research. However, APT malware differs from traditional malware in many ways [7, 8]. The advancement is evident in the highly customized malware created by APT organizations. They achieve this by combining various benign and malicious behaviors, resulting in customization, which allows them to employ different attack methods against different targets and achieve stealthiness by generating fake executable files. Moreover, compared to ordinary malware, APT malware exhibits more instances of network events and other behavioral activities, showcasing the persistence of APT malware.

At present, industrial analysis on the attribution of APT samples mainly relies on the manual analysis by safety experts, which is greatly affected by the expert experience [9]. Moreover, it cannot meet the demand for many samples, resulting in low efficiency and long processing time. In the academic field, the tracing of attack samples still relies on single features, which can be categorized into structural and behavioral features. Shen G et al. [10] employed a technique that converted the original binary file into a grayscale image. They then extracted both local and global texture features from the image. This approach aimed to capture both fine-grained details and overall structural characteristics of the data. Kida M et al. [11] used fuzzy hashing to classify the original binary file using machine learning methods. Zhang Y et al. [12] represented the opcode as a vector using n-grams and used the BinMLM model based on RNN to extract the long-term dependency of APT malware but did not consider the issue of multiple code reuse in the local code during the development of APT malware. Rosenberg I et al. [13] used DNN (deep neural network) as a classifier and trained the classifier by inputting the API sequence obtained from sandbox runtime behavior to classify APT organizations. However, the API sequence did not consider the DLL link library or the generated hidden file address during the execution of the behavior, nor did it consider the correlation between event behaviors. Compared to binary files, opcodes can reflect the instructions during software runtime. At the same time, single-feature tracing methods are easily influenced by confusion [14].

Therefore, we propose a multi-feature fusion approach that combines Opcode word frequency image features, dynamic behavior features, and deep learning techniques for effective APT malware tracing. Our objective has three parts: (1) Obtaining opcode and dynamic behavior reports from APT malware. (2) Automatically extracting corresponding features using deep learning methods. (3) Improving overall classification accuracy by building a multi-feature fusion framework.

To achieve our goals, we collected 2809 executable files from the publicly available dataset cyber-research, which includes 12 related APT organizations. Firstly, we developed an event behavior graph incorporating information such as API, DLL linkage, and file addresses to address the lack of event correlation in the API sequence. We design the graph neural networks(GNNs), which contain gated graph sequence neural networks(GGNN) [15] and graph attention network(GAT) [16] to learn the features of the event behavior graph. Secondly, compared to binary code, opcodes can better reflect the running instructions of the operating system. For the first time, we constructed an opcode word frequency image to simplify the analysis process of the opcode. We used CNN-LSTM(ImageCNTM) to learn the image’s local spatial correlation and continuous long-term dependency relationship. Finally, as single features can easily be influenced by confusion, the interaction between opcode structure features and behavior features can reflect the basic operations of disassembled opcode instructions and operating systems interactions, as well as operations on files, processes, registries, module loading, and networks. Therefore, we implement our deep learning-based multi-feature fusion framework by integrating the learning features from each subcomponent through a neural network classification.

The main contributions of this paper are as follows:

- Considering that APT malware is prone to confusion, we combine the advantages of structural and behavioral features with deep learning technology to introduce a more comprehensive method for analyzing APT attacker behavior. While the behavior tends to be similar within the same APT family, we found that the proposed method, which considers opcode image features and event behavior graph features, can be used for effective classification.
- We designed the GNNs model using GGNN and GAT to learn the graph’s content features and association features. Additionally, we constructed an image based on opcode word frequency and used the ImageCNTM model to learn image features.
- The multi-feature fusion model based on deep learning demonstrated superior performance, yielding improved results. Additionally, for a single feature, the model based on opcode word frequency performed better in classification than similar papers based on structural features. Finally, we validated the effectiveness of each module in the ablation experiments.

## 2 Related works

APT attacks are complex network attacks with an obvious purpose. They gradually attack the target network through various stages, maintaining long-term access to the target [17]. With the help of APT malware, attackers can remotely control infected machines and steal sensitive information [18]. Analyzing the characteristics of malware samples enables the attribution classification for malware [19]. Given the integral role of malware in Advanced Persistent Threat (APT) attacks, the attributes exhibited by malware can indicate the characteristics of the APT attack entity. In other words, the distinctive features observed in the malware utilized by APT attackers can provide valuable insights into the traits and capabilities of the APT organization itself [20]. Malware feature extraction methods mainly include static structural feature extraction and dynamic behavior feature extraction [21].

### 2.1 Malware characteristics

During APT attacks, perpetrators frequently employ malware as a carrier. Hence, when attributing APT malware, it is necessary to engage in cognitive analysis by considering its behavioral and structural characteristics. By comprehensively understanding the behavior exhibited by the malware during execution and examining its underlying code and structure, we can gain valuable insights that aid in accurately attributing the malware to specific APT groups or threat actors. This multi-faceted approach enables a more informed and nuanced understanding of the origins and intentions behind APT attacks.

#### 2.1.1 Dynamic behavioral characteristics

It is relatively difficult to determine whether a software will execute malicious behavior. Assessing the alignment of a program’s behavior with user requirements is crucial in identifying whether it is malicious. By closely observing the program’s actions and evaluating whether they adhere to the expected behavior defined by the user, we can determine its malicious intent [22]. Dynamic behavior data is obtained with little human involvement and can be analyzed quickly through automated analysis of batch samples. Dynamic behavior data generally includes registry events (registry field), file events (file field), network events (network field), process events (process field), system events (system field), and other data [22]. There is a connection between each event, as APT attackers use different C&C servers or malicious payloads to establish multiple network events and other events to prevent the association of the same APT family when designing APT malware. Therefore, obtaining the relationship between events and the characteristics of the events themselves is very important in the attribution and classification process of APT malware. Table 1 describes the purpose and operation of each event.

**Table 1:** The purpose and operation of each event.

Behavior	Target	Operation	Purpose
*Registryevent*	Registry	-	Self-launching, collect critical information
*Fileevent*	File System	Create and read etc.	Gather and illicitly acquire vital data, compromise the system and create a backdoor entry.
*Networkevent*	Network	Network query, Transmission of data and commands	Connection to C&C servers, malicious downloads and distribution
*Processevent*	Process and thread	Create, terminate and inject	Infected systems, elevated privileges
*Systemevent*	Resources and kernel	Create, modify and shutdown	Attacking the local system and hiding your tracks to monitor the system

#### 2.1.2 Static structural features

The static structural characteristics of malware mainly include binary code features and disassembly code features [23]. Converting binary files into images [10, 24–27] has become an increasingly popular research topic. However, this method often involves compression and deformation operations such as resizing, which makes it challenging to recover resources such as import tables and export tables, resulting in information loss [28]. At the same time, because the development of APT malware involves multiple developers, some of whom may be replaced during the development process, the attribution classification of APT malware is not a conventional single-author attribution problem. In addition, assembly operation code is another common feature in static detection during development. Compared with binary code, opcode represents the operation instruction at runtime. Studies have shown that opcodes with lower frequency can better distinguish malware [29].

### 2.2 Attributional classification methods based on dynamic structural features

The attribution classification method for dynamic behavioral characteristics of APT malware primarily relies on the analysis and classification of information obtained during the execution of the malware. Rosenberg I et al. [13]used Deep Neural Network (DNN) as a classifier by obtaining behaviors during sandbox operation as input, training the classifier, and completing the classification of APT organizations. The detection accuracy of the model is 98.6%, but the experimental data samples of the test set only include samples of APT organizations from China and Russia. Wei C et al. [30] extracted the API of dynamic behavior as behavioral features, applied dynamic long short-term memory(LSTM) and attention algorithms to express data as feature vectors, and then used transfer learning to conduct multi-classification for APT families. Han et al. [8] proposed a new APT malicious software detection and cognitive framework, APTMalnsight, which extracts dynamic API information to describe behavioral features and uses machine learning methods to attribute to its respective families based on the established API sequence feature vector. Li S et al. [9] aimed at APT malicious software in the Internet of Things, pre-processed accurate dynamic behavioral data, used the TF-IDF method for feature vector representation, and designed a multi-class model based on machine learning SMOTERF to solve the problem of multi-classification and sample imbalance. Dong S et al. [31] proposed a multi-scale feature fusion method based on CNN and DNN, cleverly utilizing permission features and API call graphs. They employed DNN to learn high-level abstract representations and combined them with CNN to construct multi-scale feature representations for classification tasks. Naeem H et al. [32] proposed a deep-stacked ensemble model based on process images. This method maps process files into images and utilizes a stacked CNN network for ensemble learning, enabling the completion of detection and classification tasks.

### 2.3 Attributional classification methods based on static structural features

The attribution classification method of APT malware based on static structural characteristics mainly relies on homology analysis and organizational classification of malicious code. Shen G et al. [10] proposed a model based on a dual attention mechanism and bidirectional long short-term memory. This method performs the attribution classification using a dual attention mechanism module to extract local texture features and a bidirectional long short-term memory module to extract global texture structure features. However, this method often involves resizing and other compression and deformation operations, making it challenging to recover resources such as import and export tables, leading to information loss. Chen W et al. [33] proposed a new gene model based on a malicious software behavior knowledge graph. An APT organization gene library is obtained by filling the malware information into the gene model. The genetic similarity algorithm is used to calculate the genetic feature similarity, thereby identifying the APT organization to which the malware belongs. The experimental dataset includes 237 samples from 6 APT organizations, with an accuracy of around 85%. Laurenza G et al. [7] collected more than 2,000 training datasets belonging to different APT organizations, extracted static features of malicious software, and used machine learning technology for identification, with an accuracy of over 90%. Bolton AD et al. [34] constructed call graphs (where nodes represent subroutines and directed edges represent call relationships between subroutines), measured the similarity between graphs using a simulated annealing algorithm, and finally used a random forest classifier to predict the family to which a sample belongs. Zhang Y et al. [12] represented opcode as a vector using n-gram and put forward an RNN-based BinMLM model to extract long-term dependencies of APT malicious software, representing the coding instruction style of the development team, thereby attributing classification to the APT malicious software corresponding to the family. However, the researchers did not consider the reuse issue of local opcode.

## 3 The proposed method

**Fig 1:**
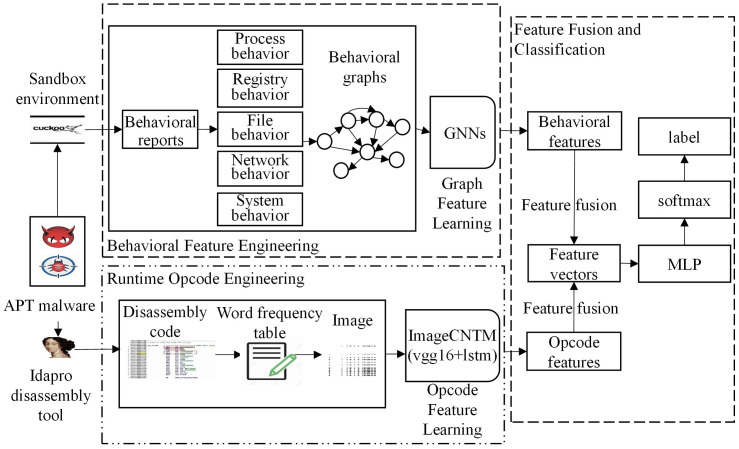
Classification framework for APT malicious software based on multi-feature fusion.

Fig 1 describes a multi-feature deep learning framework for APT malware attribution classification. The framework aims to assist security personnel. The framework mainly consists of three components:(1) Event behavior graph based on dynamic behavior, proposing a GNNs model to learn graph features automatically. Graph features reflect the advanced persistence of APT. (2) Transforming the original opcode into an easy-to-read word frequency image is beneficial for people without any security knowledge to understand, proposing a convolutional ImageCNTM model to learn image features. Opcode image features reflect the persistence of APT. (3)Opcode and dynamic behavior reports reflect the basic operations of operating system interaction and file, process, registry, system module, and network operations. Therefore, concatenating and fusing the two types of features to reflect the advanced persistence features of the overall sample. Finally, we conduct an attribution classification based on the overall features. The following sections provide an in-depth analysis of APT malicious software, data processing, and different sub-components and feature types.

### 3.1 APT malicious software feature analysis

Compared to traditional malware, APT malicious software has advanced capabilities and sustainability. Various APT organizations reflect the advanced nature of their malware through highly customized approaches and significant differences in their targets and activity behavior. They will use various means to hide their malicious behavior, such as disguising, encrypting, and self-deleting files. The persistence of APT malware manifests primarily in the frequency of network event behaviors and other event behaviors, which are notably higher compared to ordinary malware [8]. In the following sections 3.1.1 and 3.1.2, we conduct a detailed analysis of the behavior manifestations exhibited by the APT malware family.

#### 3.1.1 Analyzing the APT30 family

**Fig 2:**
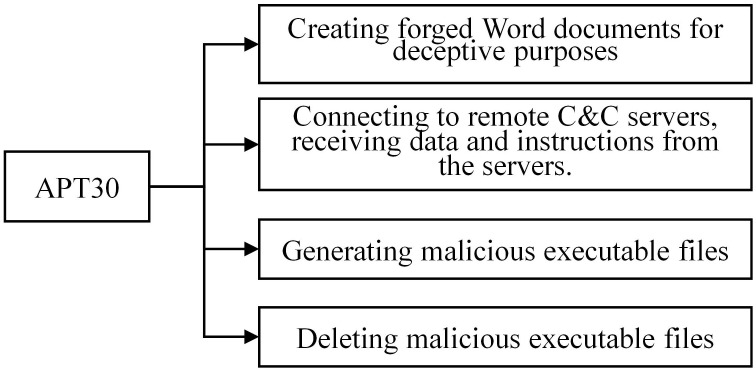
Typical malicious software behavior of the APT30 family.

**Fig 3:**
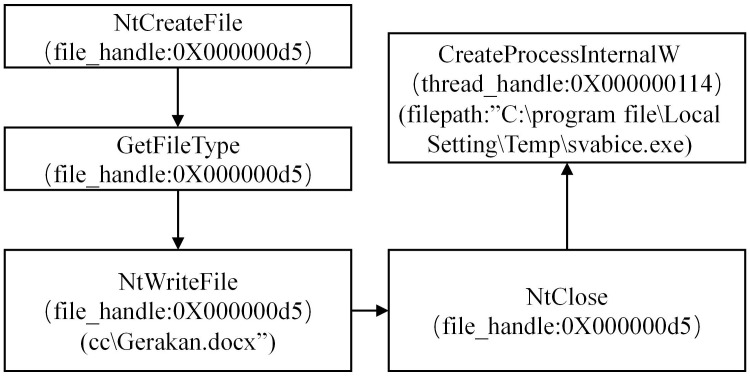
APT30 sample behavior of creating forged Word files.

**Fig 4:**
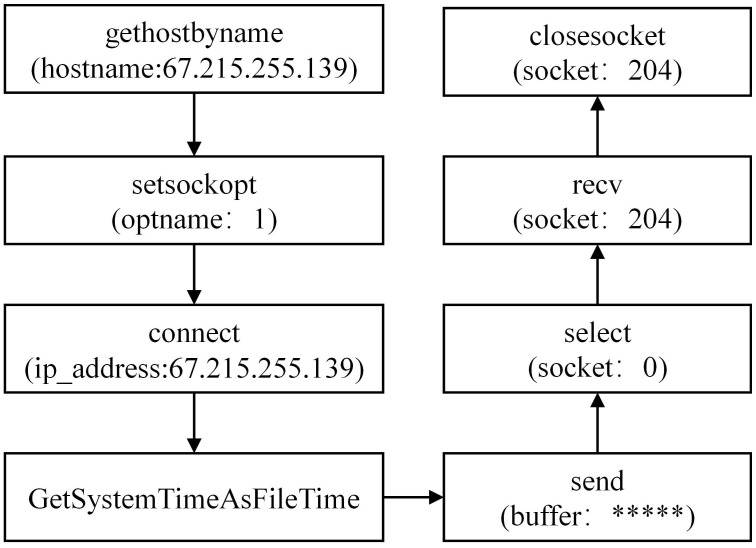
Connecting to a remote C&C server.

**Fig 5:**
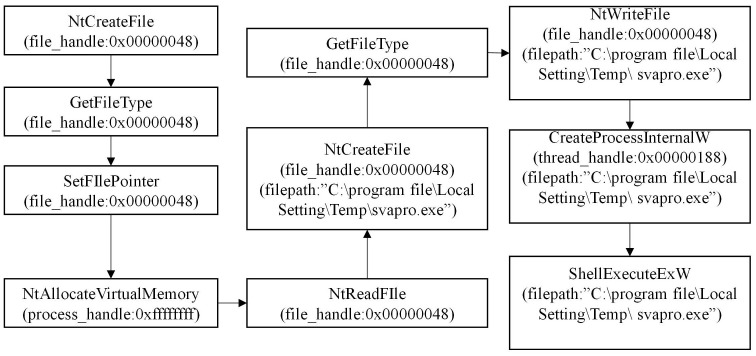
Generating malicious executable files of APT30 samples.

**Fig 6:**
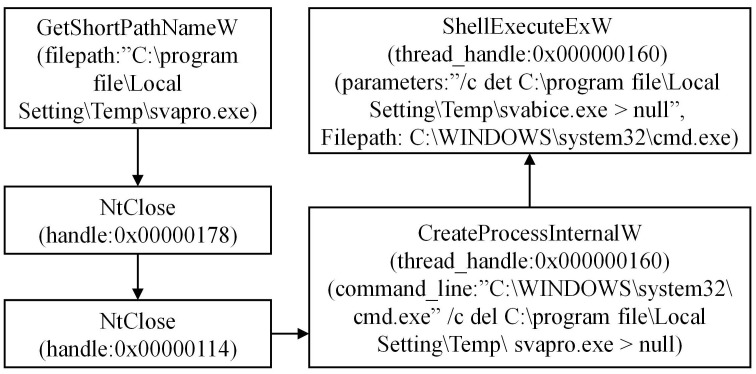
Deleting the malicious executable file generated by the APT30 sample.

During the analysis of the APT30 family, we have conducted a preliminary extraction of the attack behaviors, as depicted in Fig 2. Figs 3–6 present specific examples of each type of attack behavior. As shown in Fig 3, the attacker generates a fake Word file for deceptive purposes, which serves as a temporary storage for the results of the malicious software execution process. As shown in Fig 4, The APT30 series server establishes a remote connection to the command and control (C&C) server, enabling it to receive malicious data and commands. As shown in Fig 5, The APT30 series generates a malicious executable file named “svapro.exe” and initiates a process for executing this file. As shown in Fig 6, The APT30 family possesses the capability to self-delete, as demonstrated by its action of removing the malicious executable file “svapro.exe.” This ability allows the APT30 family to conceal its attack activities and cover up the traces of its intrusion.

#### 3.1.2 Analyzing the DarkHotel family

**Fig 7:**
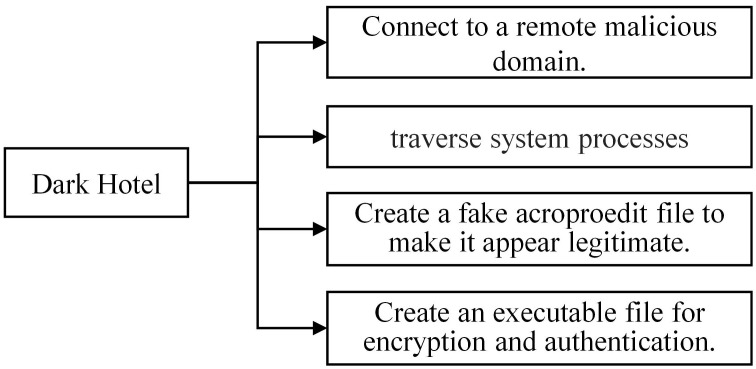
Typical malicious software behavior of the DarkHotel family.

**Fig 8:**
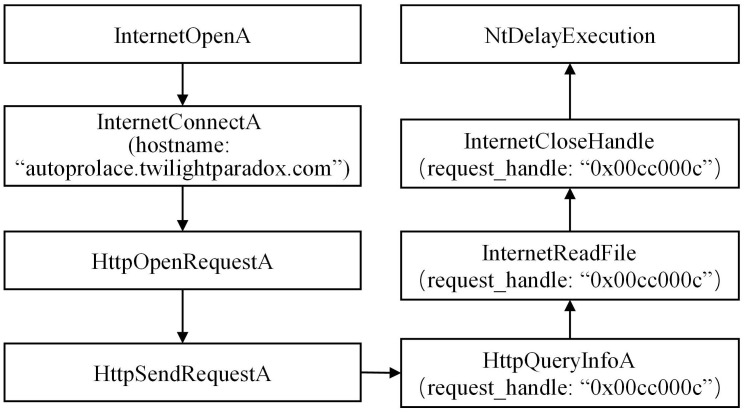
Connecting to the remote malicious domain.

**Fig 9:**
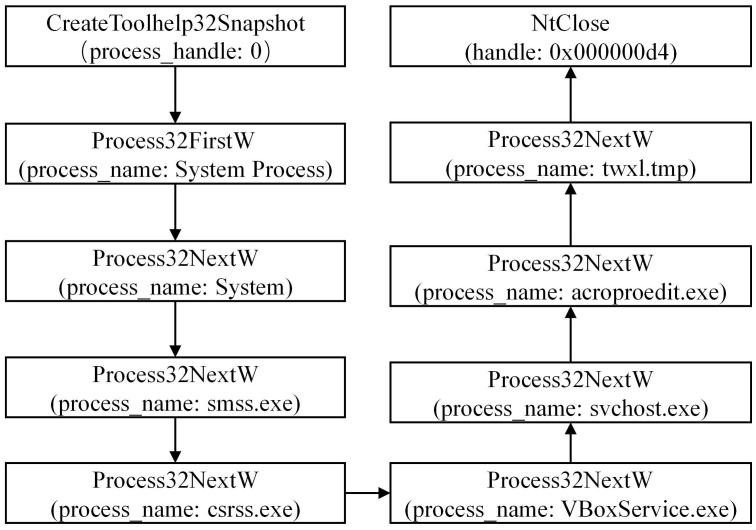
Traversing the system process list.

**Fig 10:**
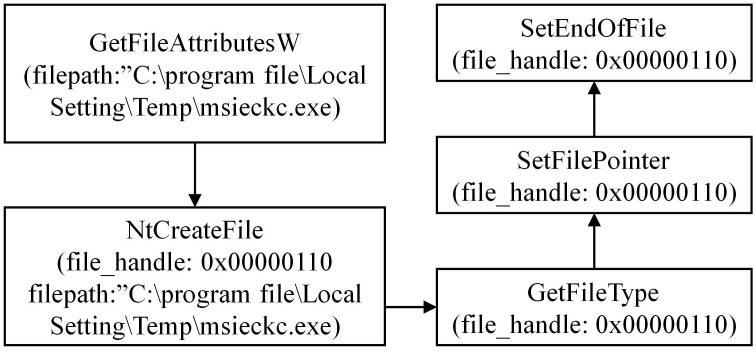
Generating a malicious executable file for encryption and authentication purposes.

**Fig 11:**
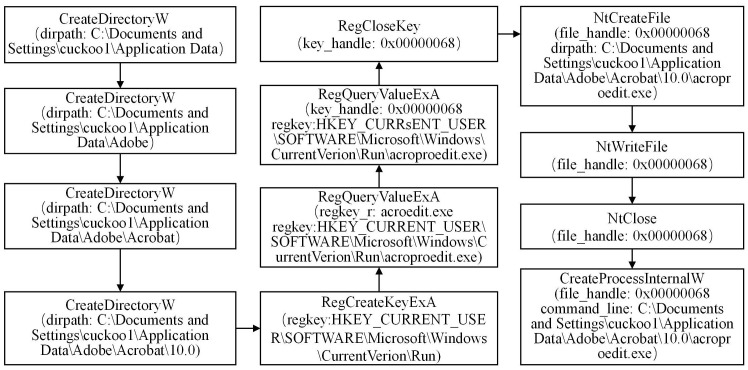
Generate a disguised acroproedit file for the Dark Hotel sample.

During the analysis of the DarkHotel family, we have conducted a preliminary extraction of the attack behaviors, as depicted in Fig 7. Specific examples of each type of attack behavior are shown in Figs 8–11. As shown in Fig 8, the DarkHotel family establishes a connection to the remote malicious domain “autoprolace.twilightparadox.com.” Upon successful connection, the malicious sample receives commands from this domain. As shown in Fig 9, The malicious sample within the DarkHotel family traverses the system’s process list to identify a specific target. Once identified, it employs a hooking technique to inject itself into the memory of the chosen process. So, the malicious sample establishes a presence within the targeted process. As shown in Fig 10, The DarkHotel family creates a malicious executable file to ensure encryption and authentication. During this process, the file undergoes encryption using the DES algorithm. The encrypted file is a container for storing and encrypting sensitive data, facilitating secure transmission, and concealing the underlying attack behavior. As shown in Fig 11, As a stealthy measure, the DarkHotel family generates a disguised file named “acroproedit” to store the data it steals from the compromised host. By adopting this disguise, the malware can elude security checks and remain hidden, thereby evading detection by the system.

### 3.2 Process event behavior graph feature

**Fig 12:**
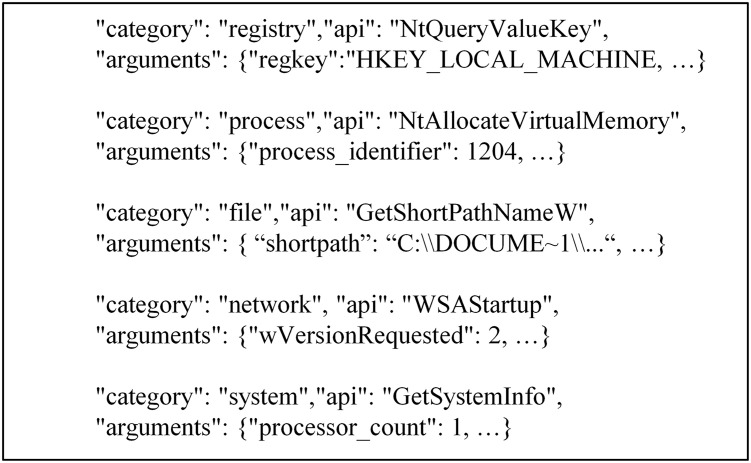
Process behavior information in json reports.

Using the upgraded cuckoo sandbox to batch-process original samples, we get a runtime JSON report. The analysis report is in JSON format, where the ‘behavior’ field contains general and process behavior. The process behavior includes registry events (registry field), file events (file field), network events (network field), process events (process field), system events (system field), etc. Fig 12 shows the process behavior information in the JSON report.

APT malware often uses data mining and theft techniques with different timelines to hide their abnormal behavior during the attack. Due to the advanced nature of APT malware, APT organizations often evade detection by combining benign and malicious behaviors to create a new way of attack and generate fake executable files for attack to achieve stealthiness. Regarding the sustainability of APT malware, there will be more event behavior occurrences and interactions between them. Traditional feature extraction methods are inadequate in extracting features representing malicious software behavior. Studies [4, 7] also point out that one of the significant difficulties in detecting APT attacks, compared to other attack techniques, is the lack of correlation in attack events [35]. Therefore, existing methods lack the behavior of APT malware and sufficient correlation in these behavioral manifestations. This paper constructs a process event behavior graph for APT malware to address these issues.

#### 3.2.1 Construct the graph

**Fig 13:**
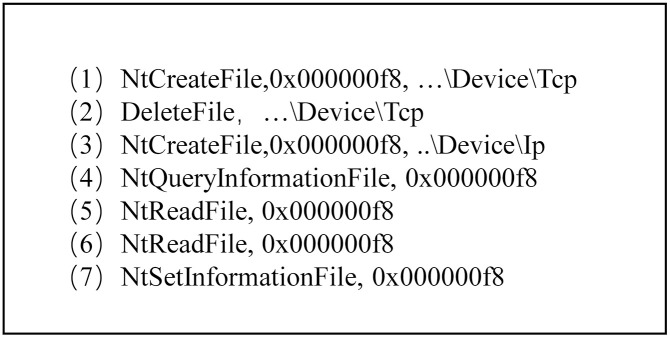
APT malware code snippet.

**Fig 14:**
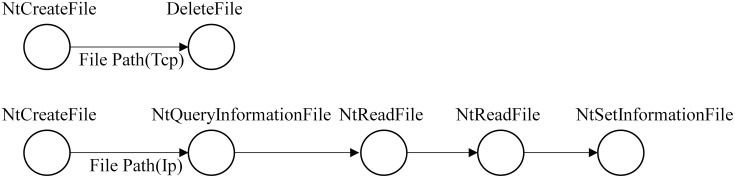
The behavior graph of the code snippet.

**Fig 15:**
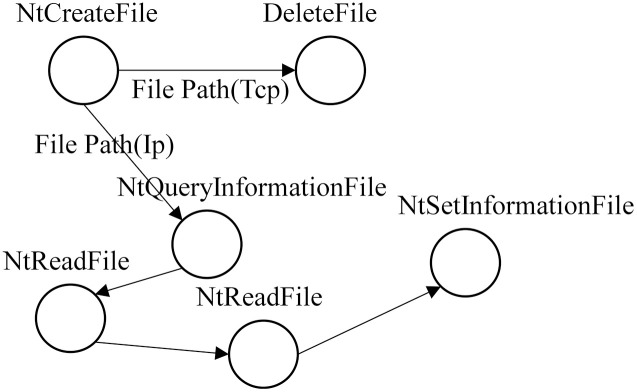
Directed multi-edge behavior isomorphism graph.

Fig 12 shows that each event in the process behavior exists independently, the API name in the event is unique, and some information in the parameter list can represent the call relationship between the events and the actions that occur in the events. Table 2 lists some connections between relevant parameters and API calls. The API calls listed in Table 2 are prone to occur in benign samples, but malicious samples may be designed if combined. For example, the Advanced Persistent Threat (APT) malicious software code snippet shown in Fig 13: (1) Firstly, the TCP file is created and then deleted (lines (1)-(2)). (2) Create an IP file. Get information, read, read again, and set IP file information (lines (3)-(7)). Fig 14 displays the behavior graph of the code snippet obtained according to the execution sequence. However, some nodes in Fig 14 are redundant. Therefore, we can merge these redundant nodes to get a directed multi-graph of behavior homomorphisms. As shown in Fig 15, it displays the specific behavior homomorphism graph.

**Table 2:** Operating system resource types and API calls.

Resource types	API calls
*Registryevent*	RegOpenKey, RegSetValue, RegCloseKey, RegDeleteValue, RegQueryValue, RegCreateKey, NtOpenKey, NtQueryValueKey, RegEnumValue, RegEnumKey, NtQueryKey, RegQueryInfoKey
*Fileevent*	NtCreateFile, NtReadFile, NtSetInformationFile, NtOpenFile, NtWriteFile, DeviceIoControl, CreateDirectory, DeleteFile, FindFirstFile, NtDeviceIoControlFile, NtQueryInformationFile
*Networkevent*	WSAStartup, getaddrinfo
*Processevent*	NtOpenSection, ZwMapViewOfSection, NtFreeVirtualMemory, NtCreateSection, CreateProcessInternal
*Systemevent*	NtDelayExecution, FindWindow, SetWindowsHook, RemoveDirectory, GetSystemMetrics, LookupPrivilegeValue

Therefore, to construct an isomorphism graph, as shown in Fig 15, the API is taken as a node, where the API name represents the node information. The parameters serve as the behavior actions connecting two events, where the parameters mainly consider DLLs, IPs, URLs, paths, and registries. The sequence of their execution forms the relationship between events. The behavior graph we propose is defined as follows:

$$\begin{array}{c} G=(V,E,\;A_{V},A_{E}) \end{array}$$ (1)

Where V represents the set of nodes, *E* ⊆ *V* × *V* represents the set of edges, and ${\tilde{A}}_{V}$ represents the node features, i.e., the API name. ${\tilde{A}}_{E}$ represents the edge features, i.e., the behavior actions (including DLLs, IPs, URLs, PATHs, and REGISTRY parameters). Algorithm 1 below describes the construction process of nodes and edges.

**Algorithm 1** Construct behavior graph from JSON report.

**Input:** report.json

**Output:** Behavior Graph

1: events ← initialize_Event(R)

2: api2Node_dir ={}

3: Node2api_dir = {Node_list=[], item_list=[],

4:        edge_list=[], attr_edge=[]}

5: num = 0

6: **for** *i* ← 0 to |events| **do**

7:  event ← get_parameter(event[i])

8:  **if** event.api ∉ Node_set **then**

9:   Node_set.add(event.api)

10:   api2Node_dir[event.api] = num

11:   *Node*2*api*_*dir*[num]←event.api

12:   num ← num + 1

13:  attr_edge.add(event.information)

14: **for** *i* ← 0 to |events| **do**

15:  key ← api2Node [event.api] item_list.add(key)

16: **for** *i* ← 0 to |item_list| **do** edge_list.add(item_list[i], item_list[i+1])

17: **return** construt_graph={Node_list, edge_list, Node2api_dir, attr_edge}

#### 3.2.2 Graph encoding

The API name is a string that represents a system call sequence with semantic relationships in context. Therefore, We utilized Word2Vec with a skip-gram model in this paper to generate a fixed-size vector space. Our training involved a substantial corpus of API calls. Following training, we transformed each unique API name into a feature vector of 32 dimensions. During the event analysis, the behavioral parameters were the file paths, dynamic link libraries (DLLs), registry entries, URLs, and IP addresses. We considered these parameters vital to capture the analyzed events’ behavioral characteristics. Since these parameters are addresses and paths represented as strings, they do not have any meaningful semantic relationships. However, we observed that there are similarities between paths and between addresses. Therefore, we performed similarity encoding on the above parameter information, with each type of parameter represented as a 16-dimensional feature vector. Initially designed for high-cardinality string variables in database learning, similarity encoding [36] serves as a lightweight feature extraction technique. This method operates on the premise that strings with a significant number of shared n-grams tend to possess similar meanings or semantic relationships. By leveraging this concept, similarity encoding enables the representation of strings in a manner that captures their inherent similarities in meaning. Therefore, we use similarity encoding to express the semantic information of event parameters. The similarity encoder essentially represents string variables as semantic feature vectors. Given a training corpus C containing N strings, the similarity encoder workflow can be dissected into three distinct steps:

(1) Definition of the similarity function. The similarity function is:

$$\begin{array}{c} sim(s_{i},s_{j})=J(G(s_{i}),G(s_{j}))=\frac{\left|G(s_{i})\cap G(s_{j})\right|}{\left|G(s_{i})\cup G(s_{j})\right|} \end{array}$$ (2)

Where G(s) represents the set of consecutive character *n* − *grams* of the string *s*, and the similarity between *s*_*i*_ and *s*_*j*_ is equal to the *Jaccard* index between $G(s_{i})$ and $G(s_{j})$. For example, if we consider 5 − *grams*, $G\left(\prime C:/Program\;Files^{\prime }\right)={\{}^{\prime }C:/Pr^{\prime },\prime :/Pro^{\prime }{,}^{\prime }/Prog^{\prime },\cdots {,}^{\prime }\;File^{\prime }{,}^{\prime }Files^{\prime }\}$, $G\left(\prime C:/ProgramFiles{\left(X86\right)}^{\prime }\right)={\{}^{\prime }C:/Pr^{\prime },\text{"}:/Pro^{\prime }{,}^{\prime }/Prog^{\prime },\cdots {,}^{\prime }\;s(X86^{\prime }{,}^{\prime }{\left(X86\right)}^{\prime }\}$, then $\text{sim}{(}^{\prime }C:/Program\;Files^{\prime }{,}^{\prime }C:/Pro-..gram\;Files{\left(X86\right)}^{\prime })=\frac{12}{17}$, To obtain more string features, we generate string sets using 3 − *grams*, 4 − *grams*, and 5 − *grams*.

(2) Training of the encoder. The strings that appear *K* times in the corpus *C* are extracted as frequent item-sets *D* = {*d*_1_, *d*_2_, ⋯, *d*_*k*_}, where *D* ⊆ *C*, and then *D* is encoded into feature vectors.

(3) Feature encoding. The similarity function encodes the string s into a feature vector of dimension size 16.

This research paper analyzes file paths, dynamic link libraries (DLLs), registry entries, URLs, and IP addresses, as they are closely associated with process event behavior. We train a separate similarity encoder for these five string types to facilitate the encoding process, resulting in five distinct encoders. During the feature encoding phase, we employ regular expression matching to identify the string type and apply the relevant encoder to process that particular string. This approach ensures that each string is encoded appropriately based on its corresponding type, allowing for accurate data analysis and representation.

### 3.3 Opcode image feature

APT malware often uses different techniques to hide its malicious behavior during attacks. Given the persistence of APT malware, a sample file may contain multiple repetitive commands, which represent the sustainable process of the APT malware attack. At the same time, the interaction of opcode structure features and behavior features can reflect the fundamental operations of the interaction between disassembled opcode commands and the operating system, as well as operations on files, processes, registries, module loading, and networks. Therefore, we use IDA Pro and Python code to process the original samples in batch and obtain the disassembled language. We extracted the standard opcodes from the disassembled code in the initial step. These opcodes encompass a range of instructions, including data transfer, arithmetic logic, flow control, stack operations, string manipulation, floating-point operations, and more. In total, there are 64 opcodes. We referenced Zhang’s work [37] in selecting opcodes, and Table 3 shows the key opcodes that we extracted.

**Table 3:** Primary extracted opcodes.

Categories	Opcode
*Operation*	ins, cld, jl, inc, endp, mul, imul, daa, test, ret, z, dec, xor, std, jmp, lea, cmp, pop, add, in, call, push, sub, dw, mov, or, dd, xchg, shl, sbb, jb, jg, jnb, shr, not, ror, rol, fld, cli, stos, rep, sar, out, stc, rcl, sal, sti, cdq, wait, jo, fstp, cmc, cwd, fdiv, fxch, rcr, scas, outs, sidt, fchs, fistp, faddp, fdivr, jno

Then, based on these 64 opcodes, we obtained the word frequency for each sample. Research has shown that opcodes with smaller word frequencies can better represent a sample’s characteristics [29]. Therefore, we arrange all opcodes in ascending order according to the total word frequency and normalize them to the range [0, 255] using Eq 3.

$$\begin{array}{c} scaler255=round(\frac{255\times (Max(dec)-Min(dec))}{Max(dec)-Min(dec)}) \end{array}$$ (3)

**Fig 16:**
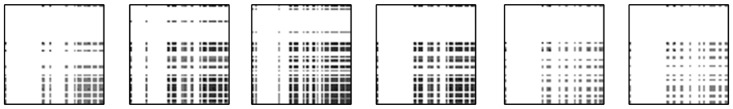
The opcode frequency co-occurrence matrix image.

Where dec represents the set of all word frequencies of the same opcode, then apply the operation 255/(*pixel* + 1) for each opcode element. We perform an analysis of adjacent operations, resulting in the derivation of a co-occurrence matrix. Subsequently, we map this matrix to an image with [64, 64] dimensions. Fig 16 displays the generated co-occurrence matrix image. The following Algorithm 2 describes the process of generating the word frequency co-occurrence matrix image.

**Algorithm 2** By using the word frequency data, we obtain a matrix image.

**Input:** asm_opcode.csv(Opcode Form Files)

**Output:** matrix image

1: data ← initialize(asm_opcode.csv)

2: api2Node_dir ={}

3: asm_opcode_cols = data.opcode

4: **for** *i* ← 0 to |asm_opcode_cols| **do**

5:  col_sum = df[col_name].sum()

6:  asm_opcode_cols.append(col_sum)

7: df[cols] = sorted(asm_opcode_cols)

8: df[cols] ← scaler255(df[cols])

9: mat[len(asm_opcode_cols)][len(asm_opcode_cols)]

10: **return** img ← img(mat)

### 3.4 Component based on event behavior graph

**Fig 17:**
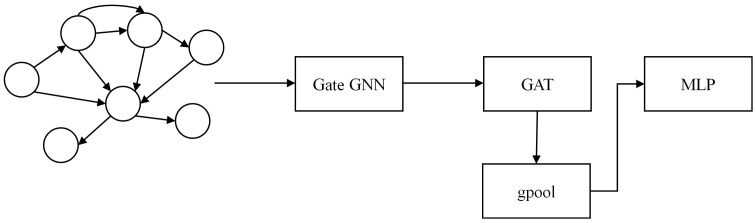
Behavior graph feature engineering module.

Dynamic events encompass registry events (“registry” field), file events (“file” field), network events (“network” field), process events (“process” field), and system events (“system” field). The intrinsic features of each event and the associated features between events are of utmost importance. Therefore, as depicted in Fig 17, GGNN [15] is employed to efficiently learn the structural information and content features, which include hidden file paths and DLL libraries. GAT+gpool learns the edge features of the graph structure and the associated features between nodes, representing the long-term persistent behavior of APT malware during its operation.

Graph neural networks operate on the fundamental principle of aggregating feature information from local graph domains using neural networks. This approach integrates neighboring node features and their relationships within the graph structure, enabling effective learning and inference processes. In recent years, researchers have developed various methods for analyzing graphs, including GGNN (Gated Graph Neural Networks) [15], GAT (Graph Attention Networks) [16], and graph convolutional networks [38], among others., based on different aggregation techniques. Due to the correlation between events and the events themselves, this paper introduces a graph neural network model (GNNs) that combines GGNN (Gated Graph Neural Networks) and GAT (Graph Attention Networks) to extract graph features. GGNN (Gated Graph Neural Networks) effectively learns the mutual dependencies between content features and nodes. GGNN (Gated Graph Neural Network) builds upon the Graph Convolutional Network (GCN) by incorporating the Gated Recurrent Unit (GRU). In this extension, the GRU considers the information from adjacent nodes as inputs, while the node’s state serves as the hidden state. The inclusion of GRU introduces selective memory of hidden information from neighboring nodes and selective memory during the iterative process of each node. This enhancement significantly improves the model’s capacity to capture and leverage contextual dependencies, leading to more effective utilization of information throughout the network. Introducing a learnable parameter W becomes essential to handle graphs consisting of nodes and edges of different types. This parameter enables the gated graph neural networks to update and propagate the embedding information of nodes and edges within graph G. Each node obtains information from its adjacent nodes through the edges and combines it with its node information during the update process. Furthermore, in addition to incorporating information from neighboring nodes, all nodes in the graph utilize the previous time series results to achieve higher-order feature interactions at time t. The formula is:

$$\begin{array}{c} a^{t}=Ah^{t-1}W_{a}+b \end{array}$$ (4)

$$\begin{array}{c} z^{t}=\sigma (W_{z}a^{t}+U_{z}h^{t-1}) \end{array}$$ (5)

$$\begin{array}{c} r^{t}=\sigma (W_{r}a^{t}+U_{r}h^{t-1}) \end{array}$$ (6)

$$\begin{array}{c} h^{t}=tanh(W_{h}a^{t}+U_{h}(r^{t}⊙h^{t-1})) \end{array}$$ (7)

$$\begin{array}{c} h^{t}=h^{t}⊙z^{t}+h^{t-1}⊙(1-z^{t}) \end{array}$$ (8)

**Fig 18:**
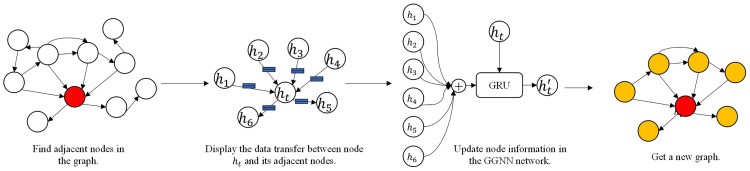
Details of operations in the GGNN network.

The initial feature values of nodes in the given equation are represented by the symbol *h*, where *d* denotes the dimensionality of the nodes. A refers to the graph’s adjacency matrix, while *sigmoid* represents the *sigmoid* function. The parameters *W*, *U*, and *b* are learnable weights and biases. *Z* denotes the update gate; the reset gate is denoted by the symbol “r” in the formula. The update gate controls the amount of new information the current state should receive from the historical state. In contrast, The reset gate actively determines the degree to which previous information is discarded for the candidate’s hidden state, making it a crucial factor in the overall process. Fig 18 provides a visual representation of the operational process, allowing for observing its intricacies and finer details.

In APT malicious samples, some events are benign, but their malicious nature becomes evident when these benign events are linked together. At the same time, in the entire behavior graph, malicious behaviors only occupy a small portion. Therefore, the contribution of behavior information from different parts of a malicious sample to the overall behavior graph varies, hence the introduction of GAT.

### 3.5 Component based on opcode image

VGG16 is a deep learning framework based on Convolutional Neural Networks (CNN) trained on the ImageNet dataset. The input to the VGG16 network architecture consists of fixed-sized images with dimensions of 224 × 224 and three color channels. The image data is then passed through a series of convolutional layers with a filter size of 3 × 3, including using 1 × 1 filters. The convolutional stride is set at 1 pixel, along with one padding, to maintain the spatial dimensions of each activation map identical to the previous layer. ReLU (Rectified Linear Units) activation functions are employed in all hidden layers to expedite the training process. Additionally, for downsampling purposes, the VGG16 network incorporates a max-pooling layer that utilizes a 2 × 2 kernel filter, has no padding, and employs a stride of 2. This layer helps reduce the spatial dimensions of the input data while retaining the most essential features. BLSTM is a bidirectional neural network that captures long-term dependencies in context by training on sequences both forwards and backwards.

**Fig 19:**
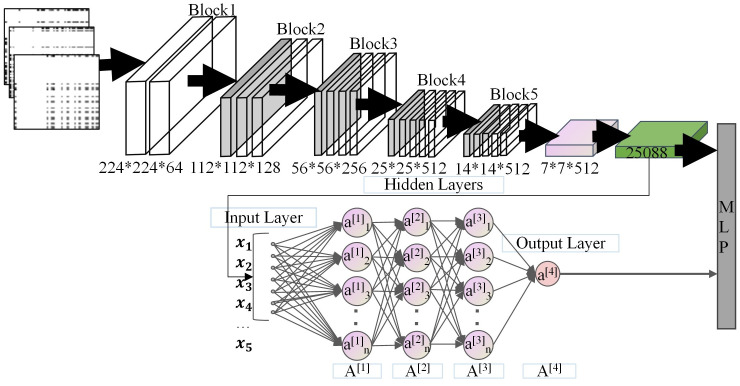
ImageCNTM model.

To address the issue of local code reuse in the same APT malware family, taking into account the simplicity of the word frequency co-occurrence matrix image and the significance of opcode frequencies, we utilize a pre-trained VGG16 model to extract local spatial features from the opcode images of disassembled language. BLSTM is used to capture the opcode sequence’s long-term dependencies, representing the malware’s persistence features. Ultimately, the paper construct the ImageCNTM model based on VGG16 and BLSTM, as shown in Fig 19.

### 3.6 Fusion component and classification

As shown in Fig 1, each sub-component in our framework extracts features from different representations, that is, different data patterns. The fusion component combines the features learned from multiple sub-components into a shared representation for the final classification.

Our approach involves several steps to ensure optimal performance before the final feature fusion and classification. First, we train each component individually, allowing them to learn specific patterns and representations. During this process, we carefully optimize the hyperparameters of each component to enhance their effectiveness. Next, we utilize the best pre-trained weights obtained from the individual training to initialize the corresponding components in the multimodal neural network. This initialization strategy helps prevent overfitting to a subset of features from a single component [39]. It also facilitates faster convergence during the subsequent training phase and improves classification results [39, 40]. This comprehensive approach ensures that each component contributes effectively to the final classification task while leveraging the advantages of pretraining and weight initialization.

#### 3.6.1 Feature fusion and classification

During training, the learned APT malware features I (based on co-occurrence matrix word frequency features) and G (behavioral graph features) are merged iteratively through multiple fusion layers. The merged feature representation serves as the shared APT malware feature. Vectors I and G are fused to form a vector P of size 8192. Subsequently, vector P is passed through nine fully connected layers and eight dropout layers for the classification of APT malware, with the last fully connected layer responsible for capturing the shared features of APT malware. The specific process is as follows:

$$\begin{array}{c} p=softmax(W_{c}P+b_{c}) \end{array}$$ (9)

Where p is a vector of size *C*(*C* = 12), *W*_*c*_ and *b*_*c*_ are the weights and biases of the layer. The *sofmax* function outputs the probability of belonging to an APT malware family’s executable file in the training set. The network configuration process defines the sizes of vectors *C* and *P*.

## 4 Experimental results

The paper wrote all the necessary code for the experiments using the PyTorch framework. The experimental environment utilizes a Windows 10 operating system with an Intel(R) Core(TM) i7-4720HQ 2.60 GHz processor, 16GB of RAM, and a 3090 graphics card. In the experiment, the paper utilized APT malware samples. The paper randomly split the training and test sets in an 8:2 ratio.

### 4.1 Datasets

The paper collected 2809 standard samples of APT malware from a public environment(https://github.com/cyber-research/APTMalware), belonging to 12 different families, including APT1, APT10, APT19, APT21, APT28, APT29, APT30, Dark Hotel, Energetic Bear, Equation Group, Gorgon Group, and Winnti. The providers of APT malware used open-source threat intelligence reports from multiple vendors. The paper collected multiple threat intelligence reports from Value1, using the hash list of all files as indicators of compromise (IoCs), and obtained Value2 target samples from VirusTotal. Table 4 shows the APT malware families and the number of samples.

**Table 4:** APT family and sample size.

APT Family	Sample Size
*APT*1	387
*APT*10	238
*APT*19	23
*APT*21	78
*APT*28	151
*APT*29	269
*APT*30	164
*DarkHotel*	263
*EnergeticBear*	132
*EquationGroup*	395
*GorgonGroup*	351
*Winnti*	358

### 4.2 Evaluation index

The experiment in this article is a multi-classification experiment. Due to the imbalance of samples among different classes, precision, recall, and F1-score were chosen as evaluation indicators to assess various classifications comprehensively. Furthermore, the paper used a confusion matrix to represent the classification results. For the i-th class(1 ⩽ *i* ≤ *n*), the precision (*P*_*i*_), recall (*R*_*i*_), and F1_score (*F*_*score*_*i*_) are:

$$\begin{array}{c} P_{i}=\frac{c_{ii}}{\sum_{j}c_{ji}} \end{array}$$ (10)

$$\begin{array}{c} R_{i}=\frac{c_{ii}}{\sum_{j}c_{ij}} \end{array}$$ (11)

$$\begin{array}{c} F\_score_{i}=2\times \frac{P_{i}\times R_{i}}{P_{i}+R_{i}} \end{array}$$ (12)

Finally, the paper calculates the arithmetic mean of the indicators for each category to obtain the macro average, which measures the overall classification performance across various algorithms.

$$\begin{array}{c} P_{macro}=\frac{1}{n}\sum_{i=1}^{n}P_{i} \end{array}$$ (13)

$$\begin{array}{c} R_{macro}=\frac{1}{n}\sum_{i=1}^{n}R_{i} \end{array}$$ (14)

$$\begin{array}{c} P\_score_{macro}=2\times \frac{P_{macro}\times R_{macro}}{P_{macro}+R_{macro}} \end{array}$$ (15)

### 4.3 Evaluating the graph-based component

Unlike traditional malware, APT (Advanced Persistent Threat) attackers use different C&C (Command and Control) servers or malicious payloads to establish network and other event behaviors to prevent association within the same APT family. Therefore, the paper constructs an event behavior graph in the dynamic behavior report by considering API calls and their corresponding event actions, such as file operations, network operations, and more. Existing methods have only used API sequences. Rosenberg I et al. [13] focused on the API sequences in the dynamic behavior report, taking the top 50,000 words with the highest frequency as behavioral features. Subsequently, they used a model based on the DNN architecture to trace the source of APT malware. Chaoxian Wei et al. [30] extracted the dynamic behavior’s API as behavioral features, applied dynamic LSTM and attention algorithms to represent the data as feature vectors, and then utilized transfer learning to perform multi-classification on APT families.

As shown in Table 5, the precision of our proposed graph neural network-based method is lower than the approach proposed by Wei C et al. [30], possibly due to incomplete behavior reports resulting from the detection of virtual environments during the simulation of sample behavior using upgraded cuckoo in the original sample, which led to incomplete construction of the behavior timeline graph and hindered the graph neural network model from better learning event-related feature correlations. However, we found that the precision was significantly higher than the method proposed by Rosenberg I et al. [13], which also demonstrates the importance of considering event and event correlation, as well as the operational details of API instructions. The paper also emphasized the significance of hidden file addresses and Dynamic Linked Libraries (DLLs) since APT malware frequently generates counterfeit executable files to achieve stealth effects. The long-term dependency between events also illustrates the sustainability of APT malware. In general, the proposed Graph-GNNs in this paper consider API instructions and related parameters, while Rosenberg et al. only consider API instructions without related parameter information. Graph-GNNs achieve an improvement of 2.82% in precision.

**Table 5:** Comparison of related papers based on dynamic behavior models.

Baseline	Evaluation Index		
	Macro P(%)	Macro Recall(%)	Macro F1(%)
Rosenberg I et al. [13]	86.42	85.29	85.11
Wei C et al. [30]	93.08	92.65	92.83
Graph-GNNs (Our Method)	**89.24**	80.65	83.15

### 4.4 Evaluating the image-based component

APT organizations involve multiple developers in the development of malware, making the structure of the original binary code diverse and increasing the difficulty of tracing. Compared to binary code, opcode can reflect software running instructions and is less likely to be obfuscated. Shen G et al. [10] focused on the malicious code itself and proposed a method for tracing APT malware based on dual attention mechanisms and bidirectional Long Short-Term Memory (LSTM) using the grayscale images of the malicious code. This method only considers the grayscale image features of the binary code, which can be easily affected by obfuscation mechanisms, leading to a decline in classification performance. Kida M et al. [11] performed fuzzy hashing on the original samples and then used machine learning methods for multi-classification tasks. Fuzzy hashing usually only compares files locally and often needs to catch up on some crucial information. Zhang Y et al. [12] performed n-gram operations on opcodes, then input them into an RNN-based BinMLM model to capture the long-term dependencies of the opcode sequence. However, they did not consider the issue of multiple reuses of local operation behavior within the same APT organization.

As shown in Table 6, the deep learning method proposed in this paper based on opcode images outperforms related papers based on binary code in all indicators, further validating that opcode instructions can better reflect software running behavior than binary code and are less likely to be affected by code obfuscation, indicating that opcode behavior instructions can reflect the sustainability of APT malware. Our proposed method outperforms the approach proposed by Zhang Y et al. [12], demonstrating the importance of local spatial features and solving the issue of multiple reuses of local operation behavior within the same APT family.

**Table 6:** Comparison of related papers based on static structural models.

Baseline	Feature	Evaluation Index		
		Macro P(%)	Macro Recall(%)	Macro F1(%)
Shen G et al. [10]	Binary code	86.25	86.02	85.89
Kida M et al. [11]	Binary code	89.23	85.37	87.35
Zhang Y et al. [12]	Opcode	91.74	88.44	89.26
ImageCNTM (Our Method)	Opcode	**91.97**	**90.61**	**91.15**

### 4.5 Evaluating multi-feature fusion model

Attribution classification based on opcode structural features or event behavior features both have their shortcomings, as attribution classification methods based on a single feature can be affected by attackers using evasion and obfuscation techniques, thereby evading detection mechanisms. Both opcode and event behavior graphs reflect the interaction and operation of the operating system, and they influence each other, both being about software running behavior. Hence, the paper compares the classification results after feature fusion with those reported in related papers.

From the experimental results in Table 7, the proposed feature fusion tracing method in this paper performs the best among all related papers, with a precision of 93.65%, recall of 93.27%, and F1 score of 93.57%. This method can partially solve the problem of APT malware being easily affected by obfuscation. Shen G. et al. [10] and Kida M et al. [11] only considered the features of the sample code itself, which we found easily disturbed by obfuscation, resulting in poor classification performance. Zhang Y et al. [12] only considered the long-term dependency relationship of opcode sequences and did not consider the problem of local code reuse of APT malware within the same family. Therefore, it performs better than methods lacking local spatial features that result in poor detection performance. Additionally, opcode behavior graphs represent the sustainability of APT malware, while event behavior graphs represent the sophistication and persistence of APT malware. Therefore, the fusion of both reflects the sophistication and persistence of APT malware.

**Table 7:** Comparison of APT malware related papers.

Baseline	Feature	Evaluation Index		
		Macro P(%)	Macro Recall(%)	Macro F1(%)
Shen G et al. [10]	Binary code	86.25	86.02	85.89
Rosenberg I et al. [13]	API	86.42	85.29	85.11
Wei C et al. [30]	API	93.08	92.65	92.83
Kida M et al. [11]	Binary code	89.23	85.37	87.35
Zhang Y et al. [12]	Opcode	91.74	88.44	89.26
This Work	Opcode-Graph	**94.23**	**93.35**	**93.72**

Rosenberg I et al. [13] and Wei C et al. [30] considered higher-level features, namely behavioral features. Rosenberg I et al. [13] focused on describing a sample’s behavior based on the top 50,000 most frequently occurring words, while Wei C et al. [30] described a sample’s behavior based on runtime API calls. API sequences pertain to runtime behavior, indicating that although dynamic execution behavior can aptly represent the software running process, it can also be affected by obfuscation and evasion techniques. So, features extracted using deep learning methods might only partially represent the complete state of the sample during its execution.

**Fig 20:**
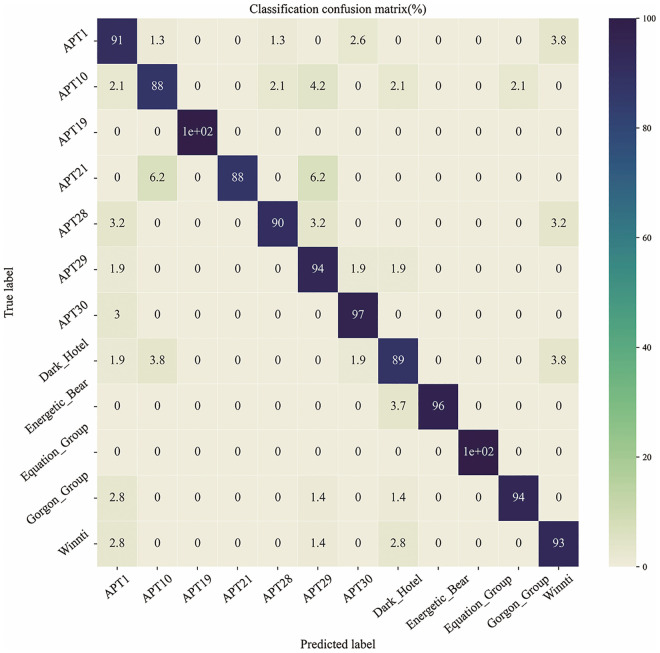
Confusion matrix for multiple classifications of APT malware.

Fig 20 shows the confusion matrix generated by the APT malware classification architecture on the APT malware testing set. The results indicate that, except for the APT10, APT21, and Dark Hotel categories, the precision of all other APT malware categories can reach over 91%.

**Fig 21:**
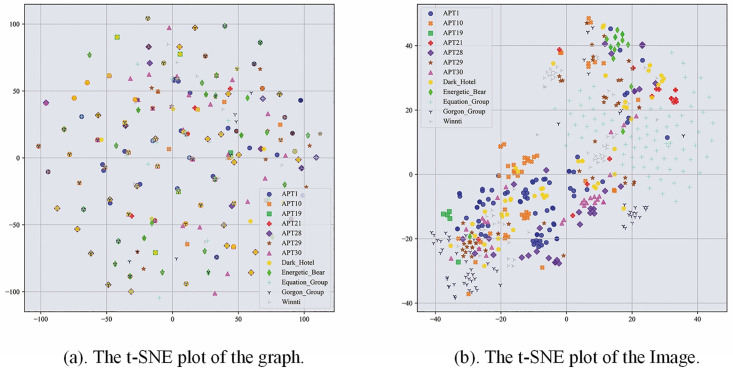
Original features.

**Fig 22:**
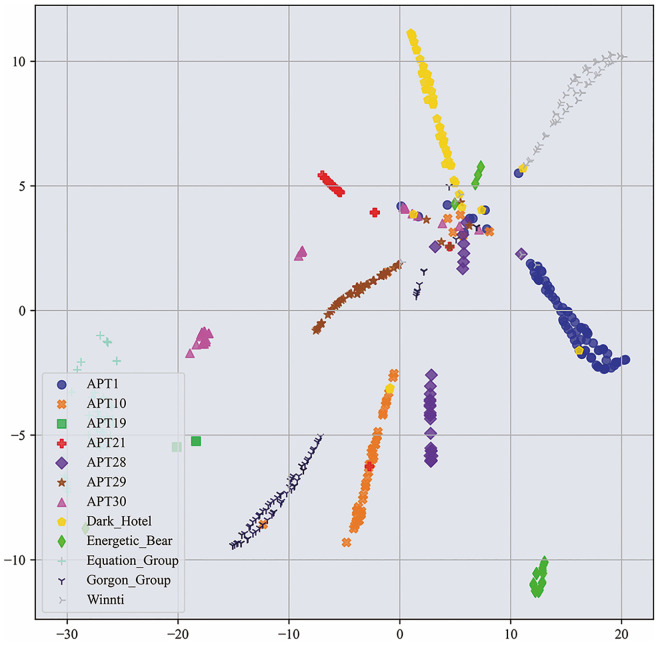
The t-SNE plot after passing through the classification layer.

In order to comprehend the source of performance improvement, this study investigates a multi-feature fusion model based on deep learning. The paper employs t-SNE (t-distributed Stochastic Neighbor Embedding) to visualize the results. t-SNE [41] is a non-linear dimensionality reduction algorithm that maps data samples to a two-dimensional space. Figs 21–22 show the visualization results. Fig 22 displays the t-SNE plot of the testing samples for the APT malware classification task. In this study, the paper utilized graphs and images as the original features of the samples. In order to visualize the sample data, the original features were projected onto a two-dimensional space, as shown in Fig 21. Lastly, the paper fused the features learned from the two types of original data and mapped the fused features to a two-dimensional space for comparison, as shown in Fig 22. The analysis revealed that the fused features formed tighter clusters than the original ones.

### 4.6 Ablation study

In this section, we constructed variants of the proposed models to evaluate the impact of the two types of features and feature fusion on the attribution classification results. The two types used were behavioral graph features and opcode image features.

The paper explored and compared the performance of different modules in the model and conducted ablation experiments from three aspects: the behavioral graph feature module, the opcode image feature module, and the multi-feature fusion module.

#### 4.6.1 Analysis of graph learning model

**Fig 23:**
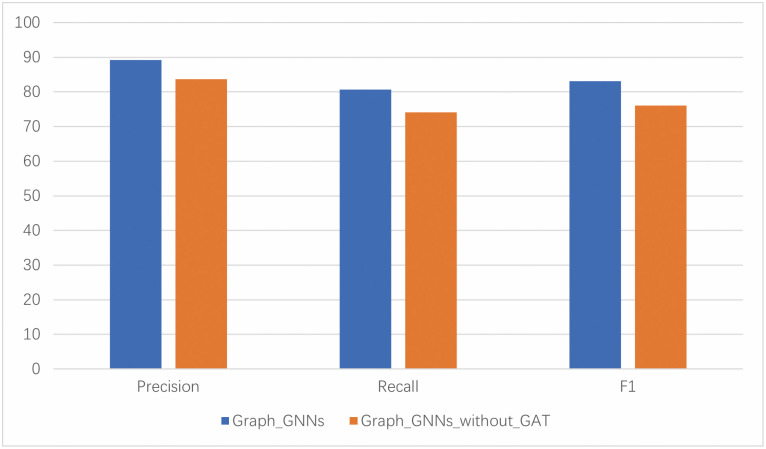
Ablation study of graph learning model.

From a technical point, the GGNN is used to capture the features of nodes and edges, which are then input into the GAT network to capture the critical features of the graph. As a comparison, section 3.4 focuses on the connection between the two. GGNN effectively learns content features and the interdependencies between nodes. On the other hand, graph attention networks(GAT) extract behavioral information from a deeper perspective. Therefore, we attempted to remove the GAT to see the impact of the key features on the performance. As shown in Fig 23, the metrics of our proposed graph neural network model (GGNN-GAT) are significantly higher than those without the GAT module, indicating that when judging the category of APT malicious samples, the contributions of different parts of behavior to the overall behavior are different. Therefore, using GAT to extract meaningful information is very crucial.

#### 4.6.2 Analysis of image learning model

**Fig 24:**
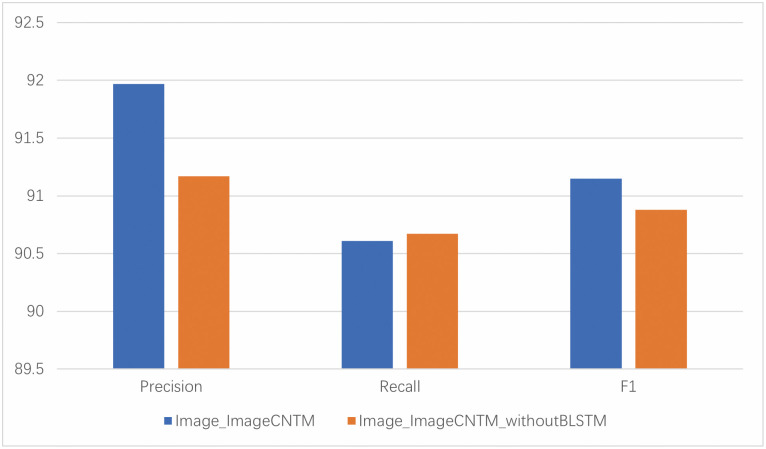
Ablation study of image learning model.

For opcode frequency, inspired by the transformation of binary code into a grayscale image, we mapped opcode frequency onto an image to get an opcode frequency image. In dealing with this image, we used the VGG16-BLSTM. Because of the influence of small samples, we used a pre-trained VGG16 to extract local features and then utilized BLSTM to extract the sequence features of the opcode. Therefore, we tried to remove BLSTM to see the impact of the continuous short-term dependency features on the performance. As shown in Fig 24, the precision and F1 score are significantly higher than after removing the BLSTM module, emphasizing the importance of the continuous long-term dependency features in the opcode image. Continuous long-term dependencies can represent a sequence of continuous operations in an opcode instruction sequence, indicating the persistence of APT malware’s actions. These operations are interrelated, interdependent, and interactive.

#### 4.6.3 Analysis of multi-feature fusion model

In this paper, we have incorporated two kinds of features: behavioral graph features and opcode image features. For these two types of features, we will merge them by removing the corresponding modules as per sections 4.6.1 and 4.6.2.

**Fig 25:**
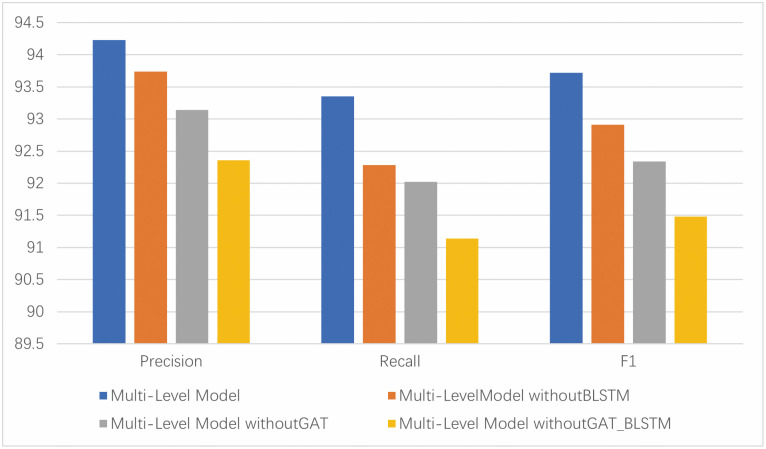
Comparison of multi-feature fusion modules.

As shown in Fig 25, the indicators of multi-stage feature fusion are significantly higher than other modules, indicating that the extraction of critical information by GAT and continuous long-term term dependency features by BLSTM are essential. For the behavior of APT malicious software samples, when determining the category of APT malicious samples, the contribution of different parts of the behavior to the overall behavior is different. Therefore, it is crucial to use GAT to extract meaningful information. For APT malicious software, continuous long-term dependencies can represent a sequence of continuous operations in an opcode instruction sequence, and there is a relationship between instructions. Therefore, it is crucial to use BLSTM to extract continuous long-term dependencies in the opcode sequence.

**Fig 26:**
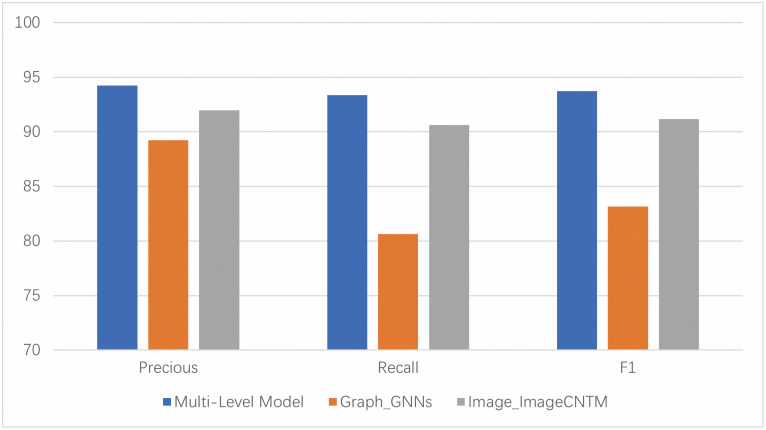
Comparison of single-feature and multi-feature fusion modules.

We considered behavioral graph features and opcode image features separately to compare the differences between multi-feature fusion and single-feature fusion methods. Each single feature still uses the original feature extraction method. We concatenated and fused each feature using a Multi-Layer Perceptron (MLP) for classification. As shown in Fig 26, the results indicate that our multi-feature fusion method has a high precision, recall, and F1 Score. Simultaneously, we found that the multi-feature fusion model based on deep learning outperforms the models based on opcode image and behavioral graph. Therefore, by proving that the multi-feature fusion deep learning model, which learns and combines malware features from various sources, can produce better classification results than the deep learning classifiers that rely on a single data feature. So the multi-feature fusion deep learning model can largely avoid the influence of confusion on the classification results. It also signifies that the features after fusion can represent the advancement and persistence of APT malicious software.

## 5 Conclusion and future work

In this paper, we use a deep learning framework to implement and evaluate a new APT malware classification method and analyze various APT organizations’ attack behaviors and methods as examples to gain a deeper understanding of APT malware and propose corresponding methods. This method combines multidimensional features extracted from the static structural opcode images and dynamic behavioral event graphs.

We utilize 2809 APT malware samples from 12 families to experimentally demonstrate (1) The effectiveness of event-related features and node and edge features, resulting in a specific improvement in accuracy (89.24%). (2) Compared to binary codes in static structures, opcodes better reflect software execution instructions and can represent the software’s behavioral features, leading to a significant increase in accuracy (91.97%). (3) Compared to traditional models relying on a single data feature, the multi-feature fusion deep learning model showed a notable improvement in accuracy (94.23%). In the future, we will research issues related to adversarial machine learning under the assumption that APT attackers manipulate data and use various techniques (static or dynamic features) to create adversarial examples to deceive detection and classification models. Although our proposed framework accurately calculates APT malware, future research must examine our multi-feature method’s robustness against adversarial deep learning techniques and evasion detection methods.
